# IDH1/IDH2 Mutations Define the Prognosis and Molecular Profiles of Patients with Gliomas: A Meta-Analysis

**DOI:** 10.1371/journal.pone.0068782

**Published:** 2013-07-22

**Authors:** Peng Zou, Haitao Xu, Pin Chen, Qing Yan, Lin Zhao, Peng Zhao, Aihua Gu

**Affiliations:** 1 Department of Neurosurgery, The First Affiliated Hospital, Nanjing Medical University, Nanjing, China; 2 School of Public Health, Nanjing Medical University, Nanjing, China; UCLA, United States of America

## Abstract

**Background:**

Isocitrate dehydrogenase isoforms 1 and 2 (IDH1 and *IDH2*) mutations have received considerable attention since the discovery of their relation with human gliomas. The predictive value of IDH1 and *IDH2* mutations in gliomas remains controversial. Here, we present the results of a meta-analysis of the associations between IDH mutations and both progression-free survival (PFS) and overall survival (OS) in gliomas. The interrelationship between the IDH mutations and *MGMT* promoter hypermethylation, *EGFR* amplification, codeletion of chromosomes 1p/19q and *TP53* gene mutation were also revealed.

**Methodology and Principal Findings:**

An electronic literature search of public databases (PubMed, Embase databases) was performed. In total, 10 articles, including 12 studies in English, with 2,190 total cases were included in the meta-analysis. The IDH mutations were frequent in WHO grade II and III glioma (59.5%) and secondary glioblastomas (63.4%) and were less frequent in primary glioblastomas (7.13%). Our study provides evidence that IDH mutations are tightly associated with *MGMT* promoter hypermethylation (*P*<0.001), 1p/19q codeletion (*P*<0.001) and *TP53* gene mutation (*P*<0.001) but are mutually exclusive with *EGFR* amplification (*P*<0.001). This meta-analysis showed that the combined hazard ratio (HR) estimate for overall survival and progression-free survival in patients with *IDH* mutations was 0.33 (95% CI: 0.25–0.42) and 0.38 (95% CI: 0.21–0.68), compared with glioma patients whose tumours harboured the wild-type *IDH*. Subgroup analyses based on tumour grade also revealed that the presence of *IDH* mutations was associated with a better outcome.

**Conclusion:**

Our study suggests that IDH mutations, which are closely linked to the genomic profile of gliomas, are potential prognostic biomarkers for gliomas.

## Introduction

Gliomas, which are the most common primary intracranial tumours, are classified as grade I to grade IV, according to the 2007 WHO Classification of Tumours of the Central Nervous System [1]. Despite advances in diagnostic and therapeutic techniques, the prognosis for most glioma patients remains dismal. Histomorphological criteria alone are not sufficient to predict the clinical outcome of gliomas. Thus, new avenues must be taken to integrate the molecular advances with the histological assessment of gliomas.

Recently, the sequencing of human gliomas has identified mutations in the isocitrate dehydrogenase 1 and 2 (*IDH1* and *IDH2*) genes [2]–[4]. *IDH* mutations are relatively glioma-specific. However, *IDH1* and *IDH2* mutations are also found in acute myeloid leukaemia (AML) [5]. The *IDH* gene mutations are found frequently in malignant gliomas and are likely to be involved in the early stage of gliomagenesis, even before *TP53* mutations or loss of 1p and 19q [6]. The IDH1 mutations occur in the highly conserved residue R132, which is in the catalytic domain, where it binds to its substrate. The mutations in IDH2 consistently occur at the analogous amino acid R172 [7], which is functionally equivalent to amino acid 132 of IDH1. *IDH1* mutations have been found in approximately 80% of grades II-III gliomas and secondary glioblastomas but have been found in less than 10% of primary glioblastomas [2], [4], [6]. The IDH2 mutations have also been described in gliomas, although at a lower frequency [4], [8]. The IDH1 and IDH2 enzymes catalyse oxidative decarboxylation of isocitrate into α-ketoglutarate (aKG), thereby reducing NADP to NADPH [9], [10]. The tumourigenic potential of a mutant IDH protein is under intense investigation. First, a heterozygous point mutation in codon 132 impairs the interaction of the enzyme with isocitrate both sterically and electrostatically, and the mutant IDH1 molecules dominantly inhibit the activity of wild-type IDH1 by forming a catalytically inactive heterodimer [11]. Second, the mutations cause reduced formation of aKG and decreased cytoplasmic levels of aKG increase levels of hypoxia-inducible factor subunit HIF-1alpha [11]–[13], a component of the hypoxia-responsive transcription factor complex that facilitates tumour angiogenesis and growth. Third, heterozygous IDH mutations confer neomorphic enzyme activity rather than inactivating the enzyme; the mutant enzyme converts aKG to 2-hydroxyglutarate (2-HG) in the process of consuming NADPH [14]. The excess accumulation of 2-HG has been shown to be associated with tumour progression and leads to an elevated risk of malignant gliomas [14], [15].

Recently, an increasing number of studies have evaluated the relative prognostic impact of IDH mutations and the clinical outcome of gliomas [16]–[25], with conflicting results due to the relatively small sample sizes in the studies. Here, we performed a meta-analysis to further clarify the prevalence of *IDH* mutations, their relationship to other genetic alterations and their impact on prognosis for glioma patients.

## Methods

### Identification of relevant studies

A comprehensive literature search of the PubMed and Embase databases (last search updated in October 2012) was conducted to identify all studies that analysed the prognostic role of *IDH* mutations in patients with gliomas. The following keywords were used in various combinations: ‘prognosis’, ‘prognostic’, ‘survival’, ‘*IDH1*’ and ‘*IDH2*’. The reference lists from the relevant original articles and review articles were also examined for additional relevant publications.

### Study eligibility

The studies eligible for inclusion in this meta-analysis had to meet the following criteria: (1) proven diagnosis of gliomas in humans; (2) evaluate the association between *IDH* mutations and the prognosis of glioma patients, e.g., progression-free survival (PFS) and overall survival (OS); (3) have a hazard ratio (HR) for OS or PFS, according to IDH mutations, either reported directly in the study or calculated from the data presented; (4) be the most recent or complete report if the same author or group reported results obtained from the same patient population in more than one article; and (5) be written in English.

Reports considered ineligible for the meta-analysis were (1) reviews; (2) case reports; (3) about the association between another marker and outcome and data for IDH was not presented; and (4) lacking key information such as hazard ratio (HR), 95% confidence interval (CI) or survival curve.

### Definitions and Data Extraction

The PFS was defined as the time interval between the date of surgery and the date of tumour progression or the end of follow-up. The OS was defined as the time interval between the date of surgery and the end of follow-up or death. The following data from all eligible publications were extracted: the first author's name, year of publication, country, patient ethnicity, sample size, tumour grade, mutations and prognostic outcomes (PFS and OS). Any discrepancies were resolved through discussion amongst the authors.

### Statistical Analysis

The correlations of *IDH1/2* mutations with *MGMT* promoter hypermethylation, *EGFR* amplification, codeletion of chromosomes 1p/19q and *TP53* gene mutation in gliomas were analysed using a two-sided χ^2^ test. To estimate the overall effects, the outcomes were calculated as hazard ratios (HRs) with their respective 95% confidence intervals (CIs). Subgroup analyses were performed according to tumour grade. The impact of *IDH1/2* mutations on survival was considered statistically significant if the 95% CI for the summary HR did not overlap 1.0. By convention, an observed HR greater than 1 implied a worse prognosis for the group with *IDH* mutations. The statistical significance of the pooled HR was determined using the *Z* test (*P*<0.05 was considered statistically significant). When HRs were not provided in a paper, the estimated value was derived from other data using the methods described by Tierney et al. [26]. Moreover, when univariate and multivariate analyses of PFS and/or OS were available, the multivariate analyses were combined because the survival response was influenced by multiple factors. The heterogeneity between the studies was tested using the *Q*-statistic. When the *Q*-test reported a *P* value greater than 0.05, the fixed-effects model (Mantel–Haenszel method) was used; otherwise, the random effects model was chosen, according to the DerSimonian–Laird method. The *I^2^*-statistic was also calculated to efficiently test heterogeneity (*I^2^*<25%, no heterogeneity; *I^2^* = 25–50%, moderate heterogeneity; and *I^2^* >50%, large or extreme heterogeneity). Finally, a funnel plot and Egger's linear regression test were used to assess the potential publication bias [27], [28]. All *P* values were two-sided. Statistical calculations were all performed using STATA version 11.0 software (Stata Corporation, College Station, TX).

## Results

### Studies included in the meta-analysis

**Figure 1:**
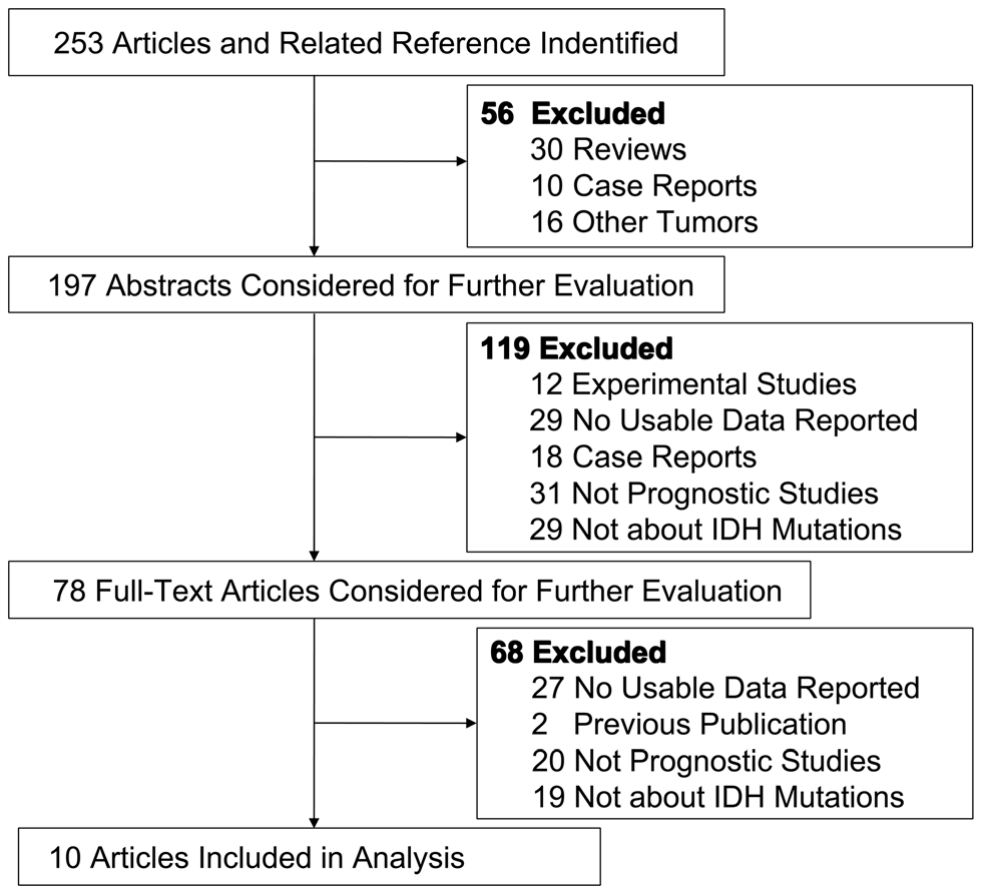
A flow chart of the study selection procedure.

Figure 1 shows the study selection procedure. By the initial literature search, 253 studies were relevant to the search terms. Of which, 175 were excluded because of obvious irrelevance by the step of screening the title and abstract. By reading through the full texts of the remaining and 68 studies were excluded (27 articles lacked usable data, 2 studies were overlapping data sets, 20 studies were not directly related to specific outcomes, 19 articles were not about IDH mutations). Overall, 10 articles, including 12 studies, published between 2009 and 2012 were used in the pooled analysis. Table 1 lists the studies and their main characteristics. In the 12 studies, the range of the sample size was 49 to 407 patients. The 12 studies collected in this meta-analysis included 6 studies on Asians and 6 studies on Caucasians. One study examined grade II tumours, four studies examined grade III tumours, four studies examined grade IV tumours, one study examined grades II-IV tumours and two studies examined tumours of all grades. An HR for PFS and OS could be extracted from 7 and 11 of the studies, respectively. All survival data were available through multivariate analysis.

**Table 1:** Characteristics of studies included in the meta-analysis.

							OS	PFS
First Author	Year	Country	Ethnicity	Case	Grade	Mutations	HR(95%CI)	HR(95%CI)
Yan	2012	China	Asian	118	IV	IDH1	0.62(0.32–1.22)	0.62(0.34–1.11)
Mukasa	2012	Japan	Asian	61	II	IDH1/2	0.33(0.07–1.53)	0.60(0.17–2.15)
Mukasa	2012	Japan	Asian	49	III	IDH1/2	0.32(0.10–0.95)	0.06(0.01–0.24)
Mukasa	2012	Japan	Asian	125	IV	IDH1/2	0.91(0.26–2.42)	0.90(0.26–2.43)
Li	2012	China	Asian	77	III	IDH1	0.15(0.04–0.66)	NA
Shibahara	2011	Japan	Asian	115	III	IDH1/2	0.16(0.07–0.37)	0.11(0.06–0.23)
Christensen	2011	America	Caucasian	131	I-IV	IDH1/2	0.27(0.10–0.72)	NA
Bleeker	2010	Netherland	Caucasian	109	IV	IDH1	0.21(0.09–0.47)	NA
Wick	2009	Germany	Caucasian	318	III	IDH1/2	NA	0.47(0.30–0.77)
Sanson	2009	France	Caucasian	404	II – IV	IDH1	0.30(0.16–0.56)	0.59(0.36–0.96)
Nobusawa	2009	Switzerland	Caucasian	407	IV	IDH1/2	0.29(0.16–0.51)	NA
Gravendeel	2009	Netherland	Caucasian	276	I-IV	IDH1	0.55(0.21–1.45)	NA

Abbreviations: OS, overall survival; PFS, progression-free survival.

### Correlation of IDH mutations with genetic aberrations and grade of the gliomas

The χ^2^ test were carried out to analyze the significance of the correlation of *IDH* mutations with other genetic alterations and glioma grade.

The frequencies of *MGMT* promoter hypermethylation, *EGFR* amplification, codeletion of chromosomes 1p/19q and *TP53* gene mutation and their relationship with *IDH* mutations are shown in Table 2. We provide evidence that *IDH* mutations are closely associated with 1p/19q codeletion (*P*<0.001), *TP53* gene mutation (*P*<0.001), and *MGMT* promoter hypermethylation (*P*<0.001), but they are mutually exclusive with *EGFR* amplification (*P*<0.001). These data indicate that the *IDH* mutation rate is linked to the genomic profile of gliomas.

**Table 2:** The association of *IDH* mutations with the genomic profile of the gliomas from the available published studies.

Parameters	*IDH* mutation/wild type									Total (%)	*P*
	Yan, 2012	Mukasa, 2012	Li, 2012	Shibahara, 2011	Christensen, 2011	Bleeker, 2010	Wick, 2009	Sanson, 2009	Nobusawa, 2009		
**Grade**											
Primary GBM	19/99	6/109	NA	NA	4/15	10/75	NA	11/172	14/363	64/833(7.13)	
Secondary GBM	NA	6/13	NA	NA	6/1	8/5	NA	10/3	22/8	52/30(63.4)	**<0.001**
Grade II, III	NA	62/110	25/22	76/39	47/22	NA	133/62	144/77	NA	487/332(59.5)	**<0.001**
**Genetic aberrations**											
*MGMT* methylated											
+	8/17	35/38	NA	69/18	NA	NA	NA	70/63	NA	182/136(57.2)	**<0.001**
−	4/48	7/52	NA	7/21	NA	NA	NA	16/45	NA	34/166(17.0)	
*EGFR* amplification											
+	9/74		NA	8/13	0/5	NA	NA	1/89	2/115	20/296(6.33)	**<0.001**
−	10/22		NA	68/26	27/28	NA	NA	154/160	29/214	288/450(39.0)	
1p19q codeletion											
+	NA	33/3	NA	30/4	NA	NA	NA	45/5	NA	108/12(90.0)	**<0.001**
−	NA	42/173	NA	46/35	NA	NA	NA	110/244	NA	198/452(30.5)	
*TP53* mutation											
+	18/66	27/29	NA	36/15	11/5	NA	NA	9/31	26/88	127/234(35.2)	**<0.001**
−	1/30	48/147	NA	40/24	16/27	NA	NA	23/55	6/243	134/526(20.3)	

NA: not available.

We found a strong correlation of *IDH* mutations with tumour grade. The *IDH* mutations were present in the majority of grades II and III glial tumours (59.5%) but were rare in primary GBM (7.13%, *P*<0.001; Table 2). A higher rate of *IDH* mutations were found in secondary GBM (63.4%) than in primary GBM (7.13%, *P*<0.001; Table 2).

### Prognostic value of IDH mutations

**Figure 2:**
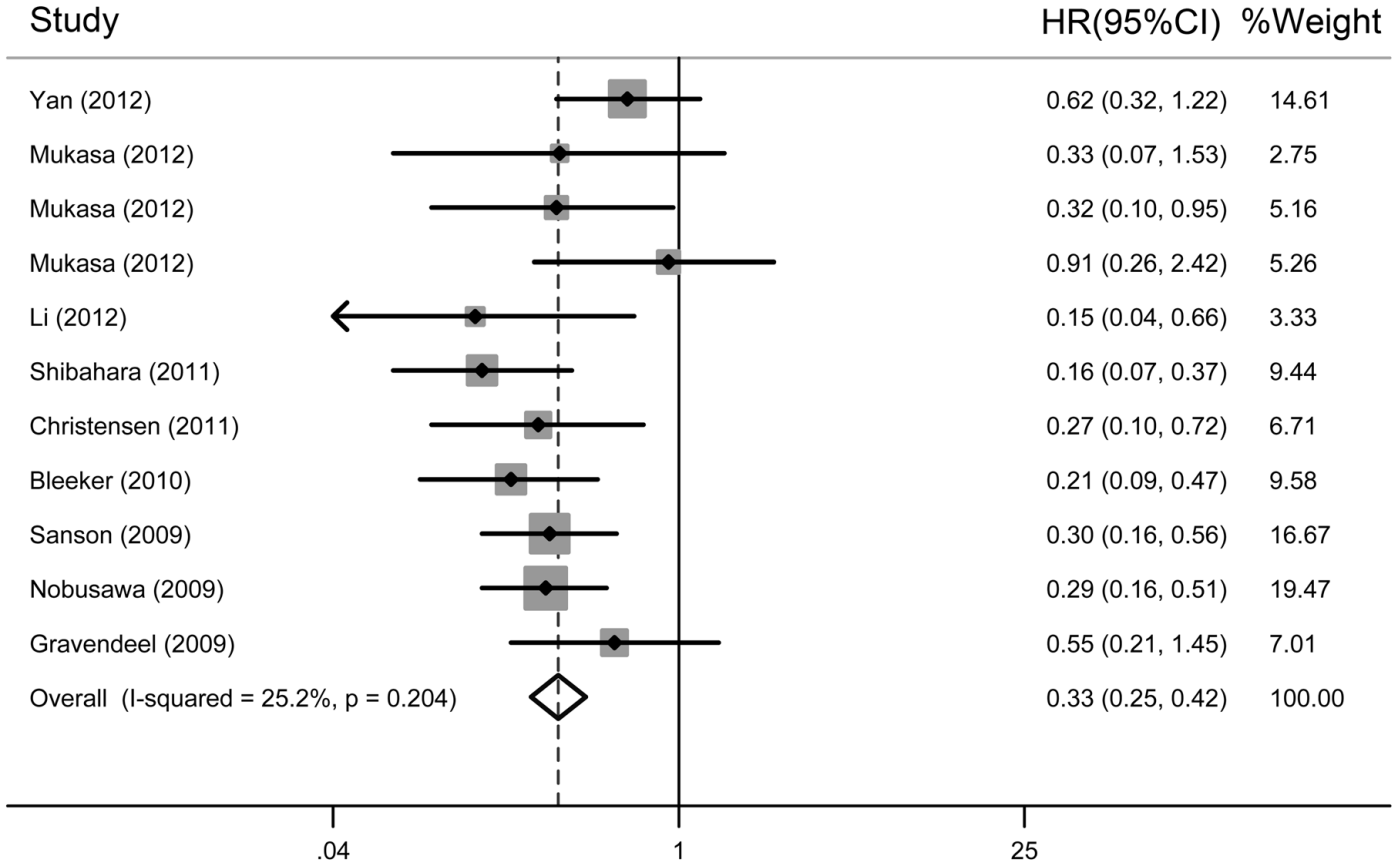
A forest plot of HR and 95% CI of the association between *IDH* mutations and OS of gliomas calculated from the multivariate Cox regression analyses.

**Figure 3:**
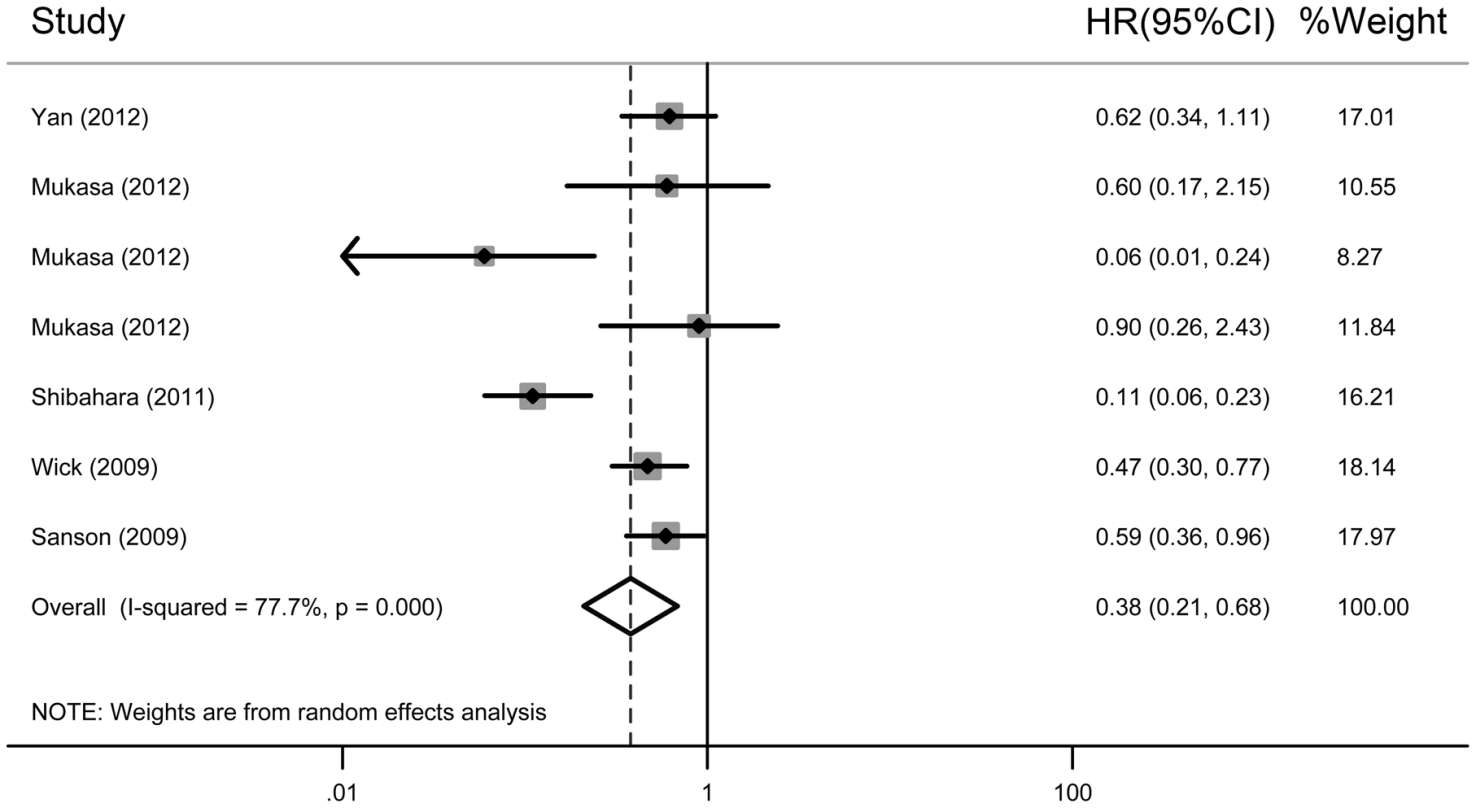
A forest plot of HR and 95% CI of the association between *IDH* mutations and PFS of gliomas calculated from the multivariate Cox regression analyses.

The pooled results of the meta-analysis showed that the *IDH* mutations were independent prognostic markers for improved OS (HR  = 0.33, 95% CI: 0.25–0.42, *P*_heterogeneity_  = 0.204; Figure 2) and PFS (HR  = 0.38, 95% CI: 0.21–0.68, *P*_heterogeneity_  = 0.000; Figure 3) in gliomas (Table 3). The subgroup analysis was performed according to tumour grade and ethnicity. In grades III and IV gliomas with *IDH* mutations, the overall HR for OS was 0.19 (95% CI: 0.11–0.35, *P*_heterogeneity_  = 0.579) and 0.39 (95% CI: 0.27–0.56, *P*_heterogeneity_  = 0.065), respectively, compared with wild-type *IDH* (Table 3). *IDH* mutations were a significant prognostic marker for PFS in grade III (HR  = 0.17, 95% CI: 0.05–0.58, *P*_heterogeneity_  = 0.000) and grade IV gliomas (HR  = 0.67, 95% CI: 0.40–1.13, *P*_heterogeneity_  = 0.000; Table 3). In Asians and Caucasians, the overall HR for OS was 0.37 (95% CI: 0.25–0.56, *P*_heterogeneity_  = 0.066) and 0.30 (95% CI: 0.21–0.41, *P*_heterogeneity_  = 0.684), respectively (Table 3). *IDH* mutations were a significant prognostic marker for PFS in Asians (HR  = 0.31, 95% CI: 0.11–0.84, *P*_heterogeneity_  = 0.000; Table 3) and Caucasians (HR  = 0.52, 95% CI: 0.37–0.74, *P*_heterogeneity_  = 0.512; Table 3).

**Table 3:** Main results of eligible studies evaluating IDH mutations and OS/PFS in gliomas.

		Heterogeneity		
	HR (95% CI)	*χ^2^*	*P* a	*I^2^*
**Overall Survival (OS)**				
**Total**	**0.33(0.25–0.42)**	13.36	0.204	25.2%
Grade III	**0.19(0.11–0.35)**	1.09	0.579	0.00%
Grade IV	**0.39(0.27–0.56)**	7.22	0.065	58.4%
Asian	**0.37(0.25–0.56)**	10.36	0.066	51.7%
Caucasian	**0.30(0.21–0.41)**	2.28	0.684	0.00%
**Progression-free survival (PFS)**				
**Total**	**0.38(0.21–0.68)** b	26.85	0.000	77.7%
Grade III	**0.17(0.05–0.58)** b	15.61	0.000	87.2%
Grade IV	**0.67(0.40–1.13)**	0.33	0.563	0.0%
Asian	**0.31(0.11–0.84)** b	23.00	0.000	82.6%
Caucasian	**0.52(0.37–0.74)**	0.43	0.512	0.0%

a *P* value of Q-test for heterogeneity test.

b Random-effects model was used when *P* value for heterogeneity test <0.05; otherwise, fix-effects model was used.

### Test of Heterogeneity and Sensitivity Analyses

Significant heterogeneity existed in the associations between *IDH* mutations and PFS (*P*_heterogeneity_  = 0.000, *I^2^* = 87.2%). However, stratification based on the glioma grade reduced the heterogeneity in the grade 4 subgroups (*P*_heterogeneity_  = 0.563, *I^2^* = 0.0%).

In the sensitivity analysis, no single study influenced the pooled HR qualitatively, which suggests that the results of our meta-analysis are stable.

### Publication Bias

**Figure 4:**
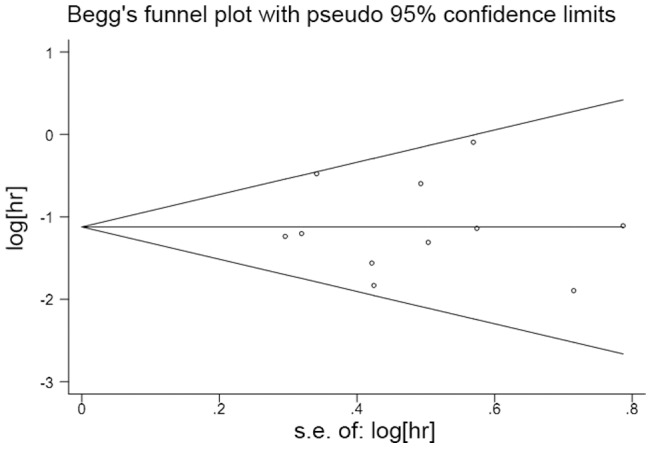
A Begg's funnel plot for the publication bias test of the *IDH* mutations and OS of human gliomas.

**Figure 5:**
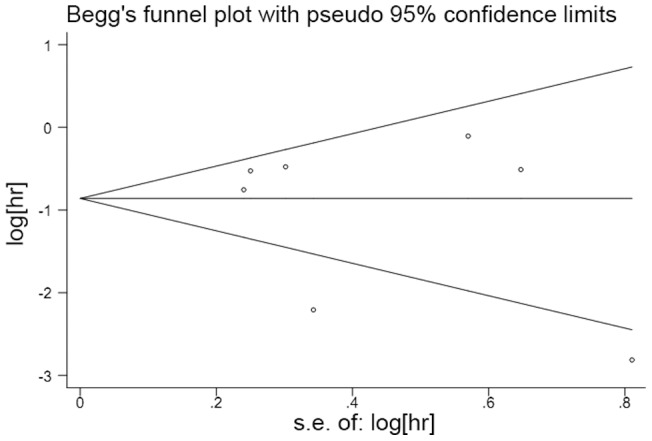
A Begg's funnel plot for the publication bias test of the *IDH* mutations and PFS of human gliomas.

We used Begg's funnel plot and Egger's test to assess the publication bias in the meta-analysis. In all studies, no funnel plot asymmetry was found. The results of the Egger's test did not show any evidence of publication bias (*P* = 0.939 for OS, *P* = 0.543 for PFS; Figure 4 and 5).

## Discussion

The last decade has observed a remarkable increase in new molecular markers that are proving to be useful as potential prognostic and predictive markers. The detection of *IDH* mutations during exome-wide sequencing of glioblastomas represents a milestone in cancer biology http://www.plosone.org/article/info%3Adoi%2F10.1371%2Fjournal.pone.0032764-pone.0032764-Parsons1#pone.0032764-Parsons1. *IDH* mutations have an important role in many aspects of gliomas, including gliomagenesis and patient prognosis. IDH1 is localised to the cytoplasm and peroxisome, whereas IDH2 resides in the mitochondria. The *IDH* genes encode redox enzymes that decarboxylate isocitrate to α-ketoglutarate (aKG), resulting in the production of NADPH and participation in cellular metabolic processes such as glucose sensing, lipid metabolism, and oxidative respiration [10], [29]. The mutated IDH have a strongly decreased enzymatic activity, leading to lower aKG production, thereby increasing HIF-1alpha levels. In addition, IDH mutations cause a loss of native enzymatic activities and thus increase the ability to reduce α-ketoglutarate to 2-hydroxyglutarate [14]. The information on the relationship of *IDH* mutations to other genetic alterations and prognostic values is still limited. In our present study, we investigated molecular and prognostic features of gliomas with and without *IDH* mutations.

We found IDH mutations were significantly correlated with glioma grade. IDH mutations were frequent in WHO grades II and III gliomas (59.5%) and in secondary glioblastomas (63.4%), but they only occur in a small fraction of primary glioblastomas (7.13%). The low frequency of IDH mutations in the gliomas with EGFR amplification most likely accounts for the low IDH mutations rate in primary glioblastomas compared with secondary glioblastomas [2]. This meta-analysis indicated that lower-grade gliomas had a different genetic aetiology from high-grade tumours and that IDH mutations occurred early in tumour development from a stem cell that can give rise to both astrocytes and oligodendrocytes.

Our study suggested that IDH mutations were closely linked to the genomic profile of the gliomas. There were significant associations between *IDH* mutations and 1p/19q codeletion (*P*<0.001), *TP53* gene mutation (*P*<0.001) and *MGMT* promoter hypermethylation (*P*<0.001), whereas an inverse association was observed between *IDH* mutations and *EGFR* amplification (*P*<0.001). The DNA-repair enzyme MGMT removes alkyl groups from the O6 position of guanine, which is the site of several chemotherapy-induced DNA alkylations, and the epigenetic silencing of the *MGMT* gene by promoter hypermethylation is associated with diminished DNA-repair enzyme activity and increased sensitivity to alkylating agents such as nitrosourea and temozolomide [30]–[32]. In the present meta-analysis, mutated IDH were strongly correlated with a higher MGMT promoter hypermethylation. Promoter hypermethylation of the *MGMT* could explain the high percentage of the *IDH1* codon 132 G395A transition because *MGMT* promoter methylation has been demonstrated to be linked to the appearance of G to A mutations in *TP53* and K-Ras [33]–[35]. Therefore, *MGMT* promoter hypermethylation could explain the high rate of the *IDH1* codon 132 G395A transition. *EGFR* activation by amplification or mutation is one of the most frequent genetic lesions in gliomas, and higher-grade gliomas are genetically characterised by *EGFR* amplification [36]. The overexpression of EGFR has been shown to promote glioma cell motility and invasion [37]. Our meta-analysis has shown an inverse association between *IDH* mutations and *EGFR* amplification. Therefore, the low proliferation rate accompanying *IDH* mutations can explain the correlation between *IDH* mutations and a favourable prognosis in glioma patients. The tumour protein p53 responds to diverse cellular stresses to regulate target genes that induce cell cycle arrest, apoptosis, DNA repair and genome stability, and p53 mutants often lead to cancer development and poor outcome [38]. *TP53* mutations are one of the most crucial factors in the development of malignant gliomas [39]. Considering the *IDH* mutations correlated with mutant P53 protein, the inherent mechanism of a better prognosis for patients with *IDH* mutations requires further investigation. Co-deletion of chromosome 1p/19q, which is commonly observed in oligodendroglial tumours, is associated with a good prognosis and increased responsiveness to chemotherapy [13], [40]. These genetic changes often occur in a staged order during malignant transformation. Watanabe et al. [6] dissected multiple biopsies from the same glioma patients and found that there was no case in which *IDH* mutations had occurred after the acquisition of either a *TP53* mutation or 1p/19q codeletion, suggesting that *IDH* mutations were early events occurring during human gliomagenesis and may affect a common glial precursor cell population.

Our meta-analysis have found that *IDH* mutations carry a very strong prognostic significance for PFS and OS. Subgroup analyses according to tumour grade also revealed that the presence of IDH mutations was associated with a better outcome. For patients with IDH mutations, longer OS was observed in patients with grades III and IV gliomas. The PFS in patients with mutated IDH and grades III or IV gliomas had a better prognosis, but this observation had no statistical significance in grade IV gliomas. In our meta-analysis all the survival data were available in the form of a multivariate analysis. Therefore, *IDH* mutations seem to be an independent favorable prognostic marker in glioma patients. The reasons for an improved outcome could potentially be related to the biological results of mutant IDH. First, mutant IDH1*^R132H^* overexpression in stably transfected glioma cell lines in vitro resulted in a marked decrease in proliferation rates, decreased Akt phosphorylation, altered morphology, and a more contact-dependent cell migration. The reduced proliferation is a consequence of the D-2-HG produced by IDH1*^R132H^*. Mice injected with IDH1*^R132H–GFP^*-expressing cells have prolonged survival compared to mice injected with cells expressing either IDH1*^wt–GFP^* or GFP [41]. Second, the *IDH1* codon 132 mutations consume rather than produce NADPH. NADPH plays an important role in detoxification processes and scavenging oxygen radicals; the low NADPH levels may be less resistant to irradiation and chemotherapy, thus explaining the prolonged survival of patients with mutated glioblastoma [20]. Third, the substitution of R132 with any one of the six amino acids observed in gliomas (His, Ser, Gly, Cys, Val, and Leu) may have a dramatically reduced affinity for isocitrate and dominantly inhibit wild-type IDH1 activity through the formation of catalytically inactive heterodimers, making the cell more susceptible to the oxidative stress induced by chemotherapy and radiotherapy [42].

The current meta-analysis has several limitations. First, because of limited data, we did not perform the stratification analyses with other variables. Second, the number of included studies was not sufficiently large enough for a comprehensive analysis. Therefore, a larger and well-designed study should be performed to further confirm the results.

Our findings strongly suggest that *IDH* mutations are associated with other genetic alterations and carry a very strong prognostic significance for PFS and OS. Further studies on the biological results of mutant IDH should lead to a more comprehensive understanding of the association between *IDH* mutations and their impacts on the outcome of gliomas.
